# MicroRNA-326 Functions as a Tumor Suppressor in Glioma by Targeting the Nin One Binding Protein (NOB1)

**DOI:** 10.1371/journal.pone.0068469

**Published:** 2013-07-15

**Authors:** Jingxu Zhou, Tao Xu, Yong Yan, Rong Qin, Hongxiang Wang, Xiaoping Zhang, Yan Huang, Yuhai Wang, Yicheng Lu, Da Fu, Juxiang Chen

**Affiliations:** 1 Department of Neurosurgery, Shanghai Institute of Neurosurgery, Changzheng Hospital, Second Military Medical University, Shanghai, China; 2 Department of Neurosurgery, PLA Hospital 101, Wuxi, Jiangsu, China; 3 Department of Nuclear Medicine, 10th people’s hospital, Tongji University, Shanghai, China; 4 State Key Laboratory of Genetic Engineering, Institute of Genetics, School of Life Sciences, Fudan University, Shanghai, China; 5 Key Laboratory of Stem Cell Biology, Institute of Health Sciences, Shanghai Jiao Tong University School of Medicine & Shanghai Institutes for Biological Sciences, Chinese Academy of Sciences, Shanghai, China; University of Florida, United States of America

## Abstract

Malignant glioma is the most common type of primary brain tumor in adults, characterized by rapid tumor growth and infiltration of tumor cells throughout the brain. Alterations in the activity of the 26S proteasome have been associated with malignant glioma cells, although the specific defects have not been identified. Recently, microRNA-326 (miR-326) was shown to play an important role in glioblastoma and breast cancer, but the underlying molecular mechanisms remain unclear. In the present study, the human Nin one binding protein (NOB1) was identified as a direct target of miR-326 and a potential oncogene in human glioma. Similar to NOB1 silencing by shRNA, overexpression of miR-326 in human glioma cell lines (A172 and U373) caused cell cycle arrest at the G1 phase, delayed cell proliferation and enhanced apoptosis. MiR-326 inhibited colony formation in soft agar and decreased growth of a xenograft tumor model, suggesting that miR-326 and NOB1 are required for tumorigenesis *in vitro* and *in vivo.* Furthermore, these processes were shown to involve the MAPK pathway. NOB1 overexpression in human glioma samples was detected by Affymetrix array analysis, and NOB1 mRNA and protein levels were shown to be increased in high-grade glioma compared to low-grade glioma and normal brain tissue. Furthermore, high levels of NOB1 were associated with unfavorable prognosis of glioma patients. Taken together, these results indicate that miR-326 and NOB1 may play an important role in the development of glioma.

## Introduction

Gliomas are aggressive lethal solid brain tumors arising from support cells in the central nervous system. The major histological subtypes of gliomas are astrocytomas, oligodendrogliomas and oligoastrocytomas. Astrocytomas, which amount to 80–85% of all gliomas, are graded from low (grade I–II) to high (grade III–IV) based on histopathological characteristics [1]. Grade IV astrocytomas are known as glioblastoma multiforme (GBM), which is the most common and lethal type of adult gliomas and is associated with a poor prognosis. The current standard of care for GBM patients-surgical resection followed by adjuvant radiation therapy and chemotherapy with the oral alkylating agent temozolomide. Despite intense efforts to optimize the treatment of gliomas, the median survival is only 12–15 months for patients with glioblastomas and 2–5 years for patients with anaplastic gliomas [2], [3]. The causes and progress of gliomas have been investigated extensively; however, the genetic factors involved in the development of this disease remain poorly understood [4]. MicroRNAs (miRNAs) function as regulatory molecules by inhibiting protein translation, and they play important roles in development, differentiation, cell proliferation, and apoptosis [5]. miRNAs dysregulation could drive tumorigenesis, through the roles miRNAs can adopt as tumour suppressors or oncogenes. Downregulation of miRNAs has been suggested to play a critical role in cancer progression [6], [7], [8].

MiR-326 was first identified in a study of miRNAs expressed in neurons [9]. It was also one of several miRNAs up-regulated in zebrafish embryos treated with a Notch inhibitor [10]. MiR-326 was shown to be expressed at low levels in gliomas, and forced expression of this miRNA was cytotoxic in standard glioma cell lines and in more resistant glioma stem-like cell lines. Transfection with miR-326 markedly reduced *in vivo* tumorigenicity of glioma cells in an orthotopic mouse model. Importantly, rescue experiments demonstrated that the phenotypic effects of Notch and miR-326 were each partially mediated by suppression of the other [11]. The dysregulation of miR-326 and its possible involvement in different cancers such as medulloblastoma, cholangiocarcinoma, and chronic lymphocytic leukemia have been suggested in previous studies [12], [13].

The yeast Nin one binding protein (Nob1p) is required for the biogenesis and function of the 26S proteasome and plays a role in RNA metabolism. The human ortholog of the *NOB1* gene was cloned several years ago [14]. The *NOB1* gene, encoding a 50 KDa protein consisting of a PIN (PilT amino terminus) domain and a zinc ribbon domain, is mainly expressed in the liver, lung and spleen. However, the physiological and pathological functions of NOB1 remain unclear, and its relationship with miR-326 has not been examined to date.

In this study, we show for the first time that miR-326 potently and directly regulates NOB1. Furthermore, we demonstrate that miR-326 inhibits the activation of the MAPK pathway, which is one of the core pathways in glioma, and miR-326 overexpression impaired cell viability and the invasiveness of glioma cells. Taken together, these results establish miR-326 as a regulator of NOB1 expression and MAPK pathway activity in human glioma, with potential therapeutic implications.

## Materials and Methods

### Tissue Preparation–Ethics Statement

The specimens of the glioma patients used in this study were provided by the Shanghai Institute of Neurosurgery. All patients gave written informed consent according to a study protocol that was approved by Tissue Committee and Research Ethics Board of Second Military Medical University. Normal brain tissues were obtained from 8 patients who underwent surgical resections for reasons other than malignancy, such as cerebral trauma, for whom a partial resection of normal brain tissue was required as decompression treatment for their severe head injuries to reduce increased intracranial pressure under the permission of each of the patient’s family. The pathological diagnoses of all enrolled patients were confirmed by two different pathologists, according to the WHO grading system.

### Cell Culture

HEK293T cells and human glioma cells A172, U373 and U87 obtained from the American Type Culture Collection (ATCC) were cultured in DMEM supplemented with 10% fetal bovine serum, 100 U/mL of penicillin and 100 µg/mL of streptomycin. Cells were cultured at 37°C in a humidified atmosphere of 5% CO_2_.

### Plasmids Constructs and Luciferase Reporter Assay

The 3′-untranslated region (3′-UTR) of *NOB1* and a mutation sequence were amplified by PCR using the primers that included a Bgl II restriction site on the 5′ and 3′ strands. The PCR products were inserted into the Bgl II sites of the pGL3-control vector (Promega, Shanghai, China) and identified by DNA sequencing. Therefore, the wild type plasmid was created containing the 3′-UTR of *NOB1* with complementary sequence of miR-326 (pGL3-NOB1 3′-UTR wild), and a mutant plasmid was generated containing the mutation sequence without complementary sequence of miR-326 (pGL3-NOB1 3′-UTR mut). Primer sequences were as follows:

NOB1-3′UTR wild-F, 5′-CAAGCTTAGCGAGTTCCCGCAGGCAAAT-3′

NOB1-3′-UTR wild-R, 5′-CTCTAGACATGATCTCTGGGCACAC-3′

NOB1-3′-UTR mut-F, 5′-CAAGCTTAGCGAGTTCCCGCAGGCAAAT-3′

NOB1-3′-UTR mut-R, 5′-CTCTAGACATGATCTCTTTTCACACAGC-3′

For the luciferase reporter assays, the human malignant glioma cell line U87 was seeded on 24-well plates and co-transfected using Lipofectamine 2000 (Invitrogen, CA, USA) with 100 ng/well of the resulting luciferase UTR-report vectors, 2 ng/well of pRL-CMV vector (internal control, Promega) and and 20 ng/well of miR-326 precursor molecules or control precursor (Applied Biosystems, CA, USA) following the instructions of the manufacturer. 24 hours after transfection, the cells were lysised and the relative luciferase activity was asssessed with the Dual-Luciferase Assay Reporter System (Promega, Shanghai, China). The experiments were performed independently in triplicate.

### Cell Transfection

A172, U373 and HEK293T cells were seeded in 24-well plates overnight and then transiently transfected with miR-326 precursor, control miR-326 antisense oligonucleotide or siRNA oligos using Lipofectamine 2000 (Invitrogen, CA, USA) following the instructions of the manufacturer. Precursor miRNA and control oligos were obtained from Applied Biosystems. The scrambled shRNA (stem–loop–stem structure) targeting *NOB1* sequence were designed and synthesized (*NOB1*-shRNA: AAGGTTAAGGTGAGCTCAT). At 48 hours after transfection, the effects of gene silencing were measured via western blotting and real-time PCR analysis.

### Microarray Analysis

Microarray analysis was performed as previously reported [15]. In brief, the total RNAs were extracted from 20 fresh frozen human glioma samples (8 high-grade glioma and 12 low-grade glioma) and 1 normal brain tissues, and then biotinylated and hybridized to Affymetrix U133 expression arrays prior to scanning for quantitation. The microarray data have been deposited in the Gene Expression Omnibus (GEO) (http://www.ncbi.nlm.nih.gov/geo/) and are accessible through GEO Series accession number GSE45921.

### Reverse Transcription and Real-time PCR

Total RNA from frozen tissue and cell samples was isolated using the Trizol reagent (Invitrogen) according to the manufacturer’s instructions. Total RNA (2 µg) was reverse transcribed using M-MLV Reverse Transcriptase Kit (Promega) according to the manufacturer’s protocol. Resultant cDNA (20 ng) was mixed with SYBR GreenMasterMix (BioRad) and amplified in CFX96 real-time detection system (Bio-Rad) according to the manufacturer’s protocol. Each sample runs in triplicates for each gene. Relative expression levels of *NOB1* mRNA were calculated by normalizing to the level of GAPDH mRNA by using comparative threshold cycle (ct) method, in which fold difference = 2^–(△ct of target gene–△ct of reference)^. Primers for amplification of *NOB1* mRNA were 5′-ATCTGCCCTACAAGCCTAAAC-3′ and 5′-TCCTCCTCCTCCTCCTCAC-3′. The primers for housing-keeping gene GAPDH was 5′-GAAGGTGAAGGTCGGAGTC-3′ and 5′-GAAGATGGTGATGGGATTTC-3′.

### Protein Extraction and Western Blotting

Proteins were extracted from human glioma tissues or a subconﬂuent culture of cells, and were then characterized using western blot analysis. After blocking with 5% nonfat milk in PBS-T for 1 h at room temperature. The rabbit polyclonal antibody against NOB1 (1∶10000, Abcam, Cat. #ab87151) or GAPDH (1∶5000, Santa Cruz Biotechnology) was incubated with blot overnight at 4°C. Secondary antibody conjugated with horseradish peroxidase (1∶10,000; Santa Cruz Biotechnology) was applied for 1 h at room temperature. Blots were developed using ECL (PE LifeScience).

### Cell Cycle Analysis by Flow Cytometry

Different cell cycle phases (G1, S or G2/M phase) are characterized by different DNA contents. Fluorescence dye propidium iodide (PI) (Sigma, USA) binds to DNA strongly at a ratio of 1∶1; hence the DNA contents of cell cycle phases are reflected by varying PI fluorescent intensities. Cells were serum starved for 24 h to synchronize the cells, and then the culture medium was replaced with complete medium containing growth factor. After 48 h of incubation, cells were harvested with trypsin-EDTA, washed with chilled PBS twice and fixed with 70% ethanol at −20°C for 2 h. The fixed cells were pelleted, re-suspended in PI/RNase/PBS (100 µg/mL PI and 10 µg/mL RNase A) for at least 30 min at 37°C in dark, then filtered through a nylon mesh of 400 screen meshes. 1×10^6^ cells were analyzed by a FACs caliber II sorter and Cell Quest FACS system (BD Biosciences, USA). This experiment was repeated three times and the results were averaged. No less than 10,000 cells were analyzed in each sample. The percentage of cells in G0/G1, S and G2/M phases was determined by (fluorescence-activated cell sorting) FACS Calibur flow cytometer (BD Biosciences, USA).

### Cell Proliferation Assay

BrdU and MTT (3-(4, 5-dimethylthiazol-2-yl)-2, 5-diphenyltetrazolium bromide) assay were used for cell proliferation measurement. After different transfection for 72 hr, A172 or U373 cells were given a 2-h pulse of BrdU (Sigma) at 4 mg/mL. Visualization of new DNA synthesis was revealed by indirect immunohistochemistry on adherent cultures using primary anti-BrdU antibody followed by a secondary antibody conjugated with horseradish peroxidase. The antibodies were viewed by developing them with the TMB Peroxidase substrate kit (Vector). An absorbance rate at 550 nm wavelengths was recorded using a 96-well plate reader. MTT was performed as reported [16]. Experiments were performed in triplicate.

### Analysis of Apoptosis by Annexin V-FITC and PI

The Annexin V-FITC Apoptosis Detection kit (Calbiochem) and PI (Sigma) was used to assess the apoptotic effect of miR-326. U373 cells with different treatment were suspended with a concentration of 1×10^6^ cells/mL. Cell suspension was transferred to a tube, centrifuged and washed by PBS. Then cells were resuspended in 0.5 mL cold Binding Buffer with 1.25 µL Annexin V-FITC, and incubated for 15 min at room temperature in the dark. Then centrifuge for 5 min and remove supernatant. Cells were resuspended in 0.5 mL cold Binding Buffer with 10 µL PI, incubated and analyzed by flow cytometry. The experiments were performed independently in triplicate.

### Colony Formation Assay

Briefly, 0.5 mL under layers consisting of 0.8% agar medium was prepared in 6-well plates. A172 or U373 cells with different treatment separately were trypsinized, centrifuged, resuspended in 0.4% agar medium (equal volumes of 0.8% Noble agar and culture medium), and plated onto the top agar at 200 cells per well. The cells were kept for growth for 14 days at 37°C. Colonies were visualized using cell staining Giemsa solution (Chemicon) and counted under the microscope.

### Nude Mouse Xenografts

Nude mouse xenografts were performed as previously reported [16]. Specific pathogen-free six-week-old female BALB/C-nu/nu mice were purchased from the Cancer Research Center of Shanghai and maintained under specific pathogen-free conditions in Second Military Medical University. When the female BALB/c-nu mice were 7–8 weeks of age, each mouse was inoculated with 1.5×10^7^ U373 cells transfected with miR-326 or miR-control or *NOB1* shRNA in 0.2 mL of medium subcutaneously in the forelimb, the mouse injected mock-infected cells as control. Tumor sizes were measured every three days in two dimensions using a caliper, and the volume (mm^3^) was calculated using the formula V = 0.5* larger diameter *(smaller diameter)^2^. The tumors were excised and weighed from the sacrificed mice after 21 days. All procedures involving animals were approved by the Animal Care and Use Committee in Second Military Medical University.

### Measurement of Phosphorylation of Signaling Proteins

The changes in phosphorylation of selected proteins in certain of signaling pathways were analyzed with Proteome Profiler Array kit (ARY003; R&D Systems, Minneapolis, MN) according to the manufacturer’s instructions. In brief, human A172 and U373 glioma cells were grown, and then infected with miR-326 precursor, control precursor or *NOB1*-shRNA. At the designated times, each dish was washed twice with phosphate-buffered saline and processed according to the kit protocol. Incubations with the array contained 300 ug of lysate protein. Net integrated pixel density for each spot (an average of duplicate spots after subtraction of average background density) was determined by densitometry and analyzed using Quantity One (ISBE, Sheffield, UK) software. Results were normalized to net integrated pixel density of kit-supplied internal positive controls.

### Immunohistochemical Staining and Evaluation

Immunohistochemical staining for NOB1 protein was performed on the validating set of glioma patients. Briefly, paraffin embedded slides were treated by hydrogen peroxide (H_2_O_2_) to block endogenous peroxidase activity, and then washed with ddH_2_O and PBS. Diluted Rabbit polyclonal to NOB1 (Abcam, Cat. #ab87151) was then added for protein binding at room temperature for 60 min. The slides were washed with PBS, incubated with biotinylated secondary antibody (Abcam), and treated with Immunopure Metal enhanced DAB substrate kit (Pierce, Rockford IL) according to the manufacturer’s instructions. Staining was categorized into four grades according to immunohistochemical scores. Briefly, for each slide, 10 randomly selected fields of view under a light microscope were examined for the average intensity of positive cells and then the intensity scores were assigned to each sample as follows: none (−), none/weak (+−), weak (+); intermediate (++), and strong (+++).

### Statistical Analysis

The Student’s *t*-test was used for statistical analysis in assays performed on glioma cell lines. For experiments of glioma tissue samples, relative expression levels of *NOB1* mRNA for each group {normal brain, low-grade glioma (LGG) and high-grade glioma (HGG)} were expressed as mean ± SE, the Mann-Whitney U test was used to compare the differences between groups. When studying the relationship between *NOB1* expression and patients’ prognosis, we first grouped glioma patients of all grades to those live longer than 24 months and those live less than 24 months, Mann-Whitney U test was then applied to compare the expression of *NOB1* between these two groups. Then the prognosis in low-grade glioma and high-grade glioma patients were also analyzed separately. Fisher’s exact test was used to compare the immunolabelling results of *NOB1* between high-grade and low-grade gliomas. SPSS 15.0 (SPSS Inc, Chicago, USA) was used for the statistical analysis and a significance level of P<0.05 was used to evaluate the difference between groups.

## Results

### *NOB1* is a Novel Target of miR-326

**Figure 1:**
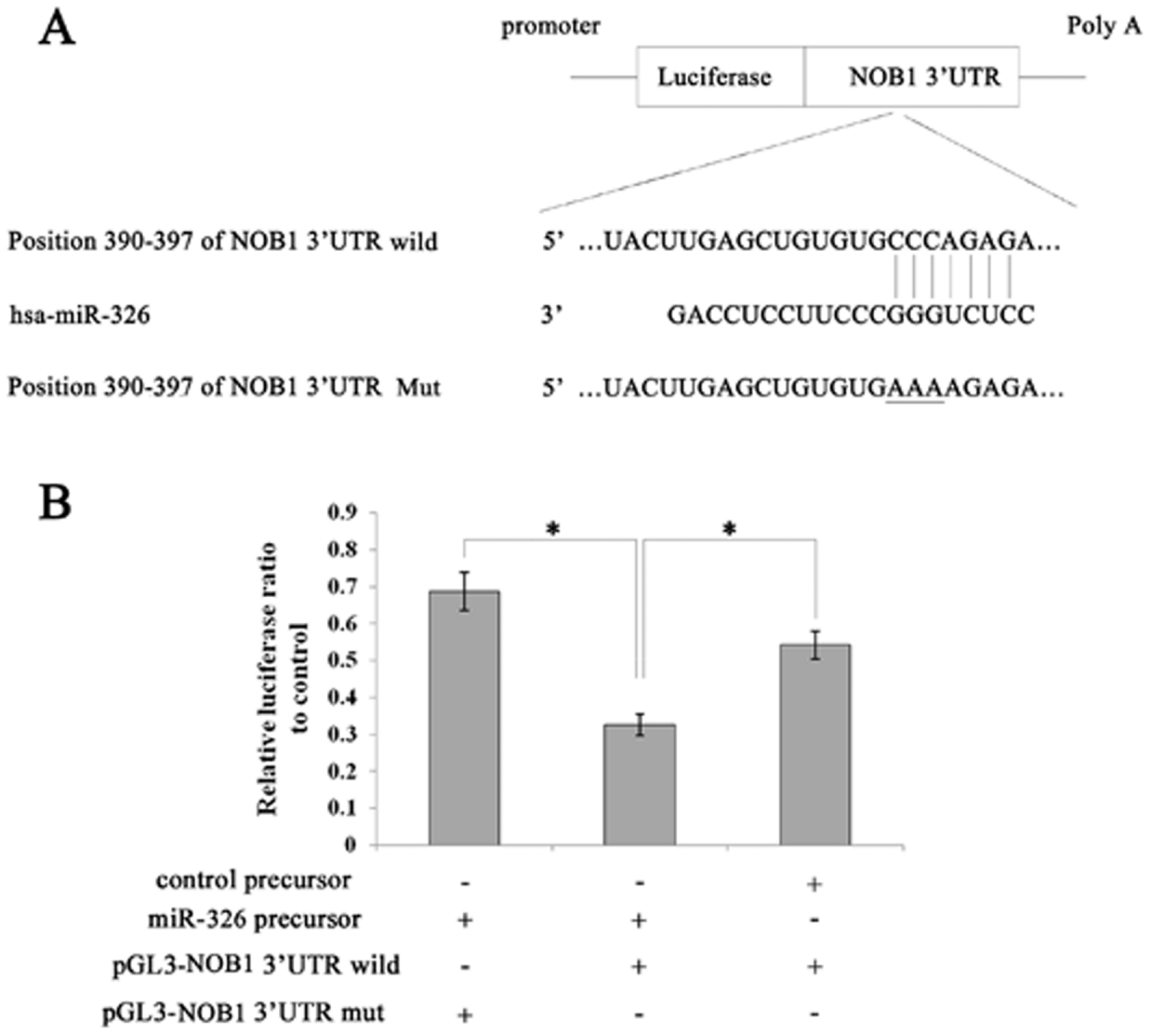
Identification of miR-326 target sites within the *NOB1* 3′-UTR. (A) Ideograph of *NOB1* mRNA. One miR-326 binding site was detected in the *NOB1* 3′-UTR. The sequence of wild-type (WT) and mutant (MT) miR-326 target sites in the *NOB1* 3′UTR are shown. A point mutation (underlined) was made in the seed region to block the binding between miR-326 and mRNA. (B) A luciferase reporter assay was used to confirm the contribution of the four miR-326 target sites. U87 cells were co-transfected with luciferase reporter plasmids containing either WT or MT miR-326 target sites and miR-326 or miR-NC precursors. miR-326 and full-length wild-type *NOB1* 3′UTR decreased luciferase activity. All results were derived from independent experiments performed in triplicate. miR-NC, non-effective control miRNA; *indicates a significant difference from the miR-NC precursor and co-transfected control plasmids (P<0.01).

Using TargetScan software [17], *NOB1* was identified as a likely target of miR-326 because it contains a putative miR-326 target site in its 3′-UTR (Figure 1A). To confirm this, a luciferase reporter vector was constructed containing oligonucleotides fully complementary to the 3′-UTR of wild-type *NOB1*, or its relevant mutant was cloned into an identical reporter vector.

Pre-hsa-miR-326 RNAs or non-functional control miR-NC RNAs were co-transfected with the above-mentioned reporter vectors into the human glioma cell line U87. The miR-326 target sequences and full-length wild-type *NOB1* 3′-UTRs reduced the relative luciferase activity only when miR-326 was present, but not when the corresponding mutant was introduced with miR-326 (Figure 1B). These results indicate that *NOB1* mRNA is a specific target of miR-326.

### MiR-326 Inhibits Glioma Cell Proliferation by Suppressing *NOB1*

**Figure 2:**
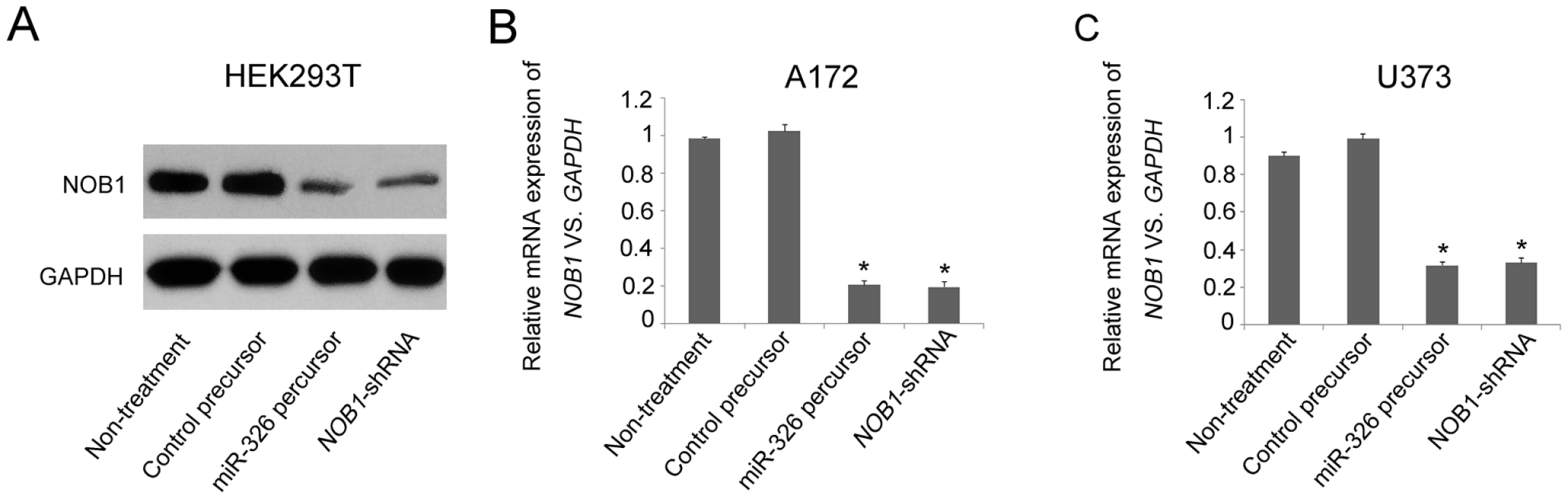
Overexpression of miR-326 down-regulated NOB1 mRNA and protein expression. (A) HEK293T cells were transfected with previously generated miR-326, miR-NC, *NOB1* shRNA or untreated control vectors. Cell lysates were separated by SDS-PAGE and transferred to a PVDF membrane, which was probed with anti-NOB1 and anti-GAPDH antibodies. miR-326 significantly repressed NOB1 protein expression (B, C) The down-regulation of *NOB1* mRNA following miR-326 transfection was determined by RT-PCR (*p<0.01).

Based on the findings described above, we hypothesized that miR-326 might inhibit cell proliferation and arrest cell cycle progression through the down-regulation of NOB1 expression. To test this hypothesis, A172 and U373 cells were transiently transfected with miR-326 or miR-NC precursors, which miR-326 significantly repressed NOB1 expression, the intervention effects of which was similar to NOB1 RNA interference (RNAi) (Figure 2).

**Figure 3:**
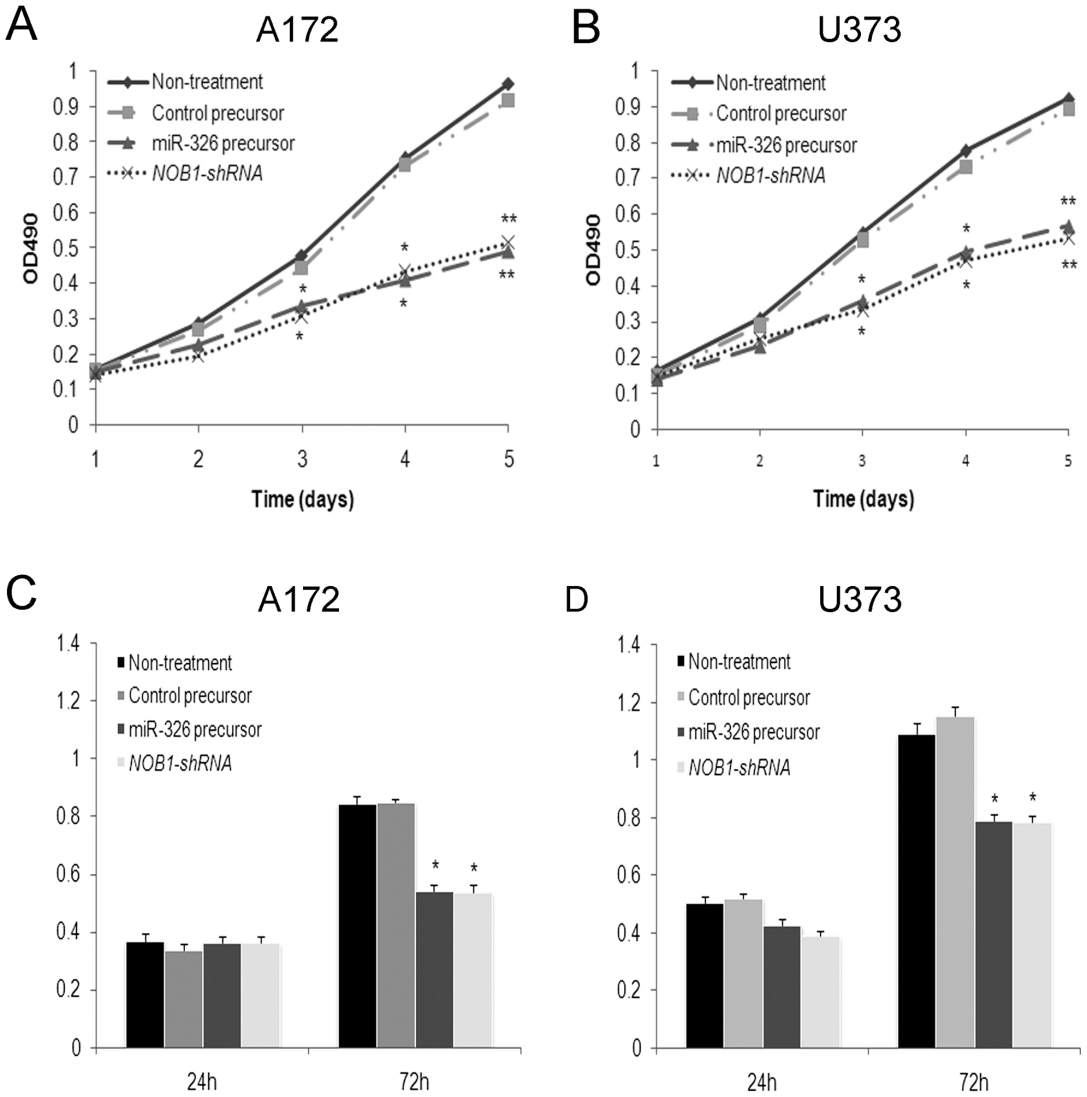
Cell viability and proliferation were examined in human glioma cells treated with miR-326 precursor. A172 (A) and U373 (B) cells were transfected with miR-326 precursor, control precursor, *NOB1* shRNA or nothing for 72 h as described in the methods section before measurement of the conversion of 3-(4,5-dimethylthiazol-2-yl)-2,5-diphenyltetrazolium bromide (MTT) to a colored formazan product. A statistically significant delay of cell proliferation was observed after day 3. A172 cells (C) and U373 cells (D) were transfected with miR-326 precursor, control precursor, *NOB1* shRNA or nothing for 72 h as described in the methods section, and the BrdU incorporation assay was performed. BrdU incorporation was decreased in the miR-326 group and *NOB1*-shRNA group compared to the controls at 72 h. Data represent the mean ± SD of three independent experiments. Significant differences between transfected cells and mock-infected cells were determined using the two-tailed Student’s *t*-test (*P<0.05, **P<0.01).

The growth of two glioma cell lines transfected with miR-326, miR-NC, *NOB1*-shRNA or untreated controls was examined using the MTT [3-(4, 5-dimethyl-2-thiazolyl) -2, 5-diphenyl -2H- tetrazolium bromide] assay. As shown in Figures 3A and B, ectopic expression of miR-326 significantly inhibited the growth of glioma cells compared to the negative control 3 d after infection (P<0.05),and there were no statistically significant differences in growth rate between miR-326 overexpressing cells and *NOB1* shRNA infected cells. After 5 d, the growth of A172 cells expressing miR-326 or treated with *NOB1* shRNA was decreased to approximately 50% of the control cells. No significant differences in cell growth were detected between miR-NC cells and the untreated controls (Figures 3A and B). The effect of miR-326 on the proliferation of human glioma cells was confirmed using a BrdU incorporation assay. No significant difference in BrdU incorporation was observed between cells with different treatment at 24 h. However, BrdU incorporation was decreased in A172 cells expressing miR-326 or through *NOB1* shRNA treatment compared to the controls at 72 h, similar results were observed in U373 cells (Figures 3C and D). Collectively, these data indicate that NOB1 may play a role in regulating the proliferation of human glioma cells.

**Figure 4:**
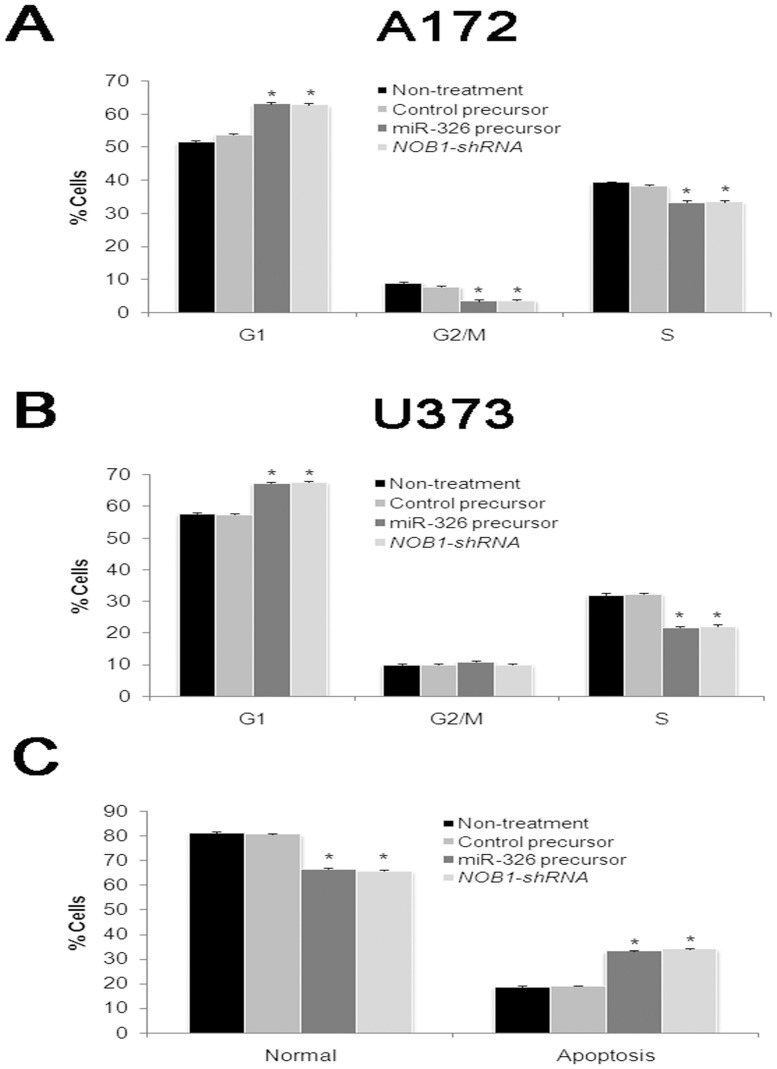
Cell cycle distribution and apoptosis of human glioma cells with decreased NOB1 *in vitro*. Percentage of cells in the G1, G2/M and S phases in A172 (A) and U373 (B) cells with different treatments as described in the methods section. Distribution patterns of normal and apoptotic cells in the four groups were determined by Annexin-PI staining(C). miR-326 expression or NOB1 silencing causes G1 cell cycle arrest, and enhanced early apoptosis. Three independent experiments were performed in each group, *P<0.05.

We then examined cell cycle distribution by fluorescence-activated cell sorting (FACS) after transfection. Compared with miR-control cells, miR-326 overexpressing cells and *NOB1* silencing cells showed a substantial decrease in the proportion of cells in S-phase and an increase in the number of cells in G1 phase in both two glioma cell lines (Figures 4A and B).

To assess the effect of miR-326 on apoptosis, human glioma U373 cells were stained with Annexin V-FITC and propidium iodide (PI) after transfection of cells with miR-326, miR-NC or *NOB1* shRNA for 72 h. As shown in Figure 4C, more than 81% of the untreated controls or miR-NC cells were viable, whereas only about 66% of the miR-326 cells or *NOB1* silencing cells were viable. Early apoptosis rates averaged from four experiments were 12.1% in the miR-NC group, 11.7% in the untreated control group 26.3% in the miR-326 group, and 27.1% in the *NOB1*-shRNA group (P<0.01). Late apoptosis rates in the four groups were approximately 7%, with no obvious differences between these groups. These findings suggest that miR-326 induced early apoptosis in glioma cells.

### Overexpression of miR-326 Inhibits Carcinogenesis in Human Glioma Cells

**Figure 5:**
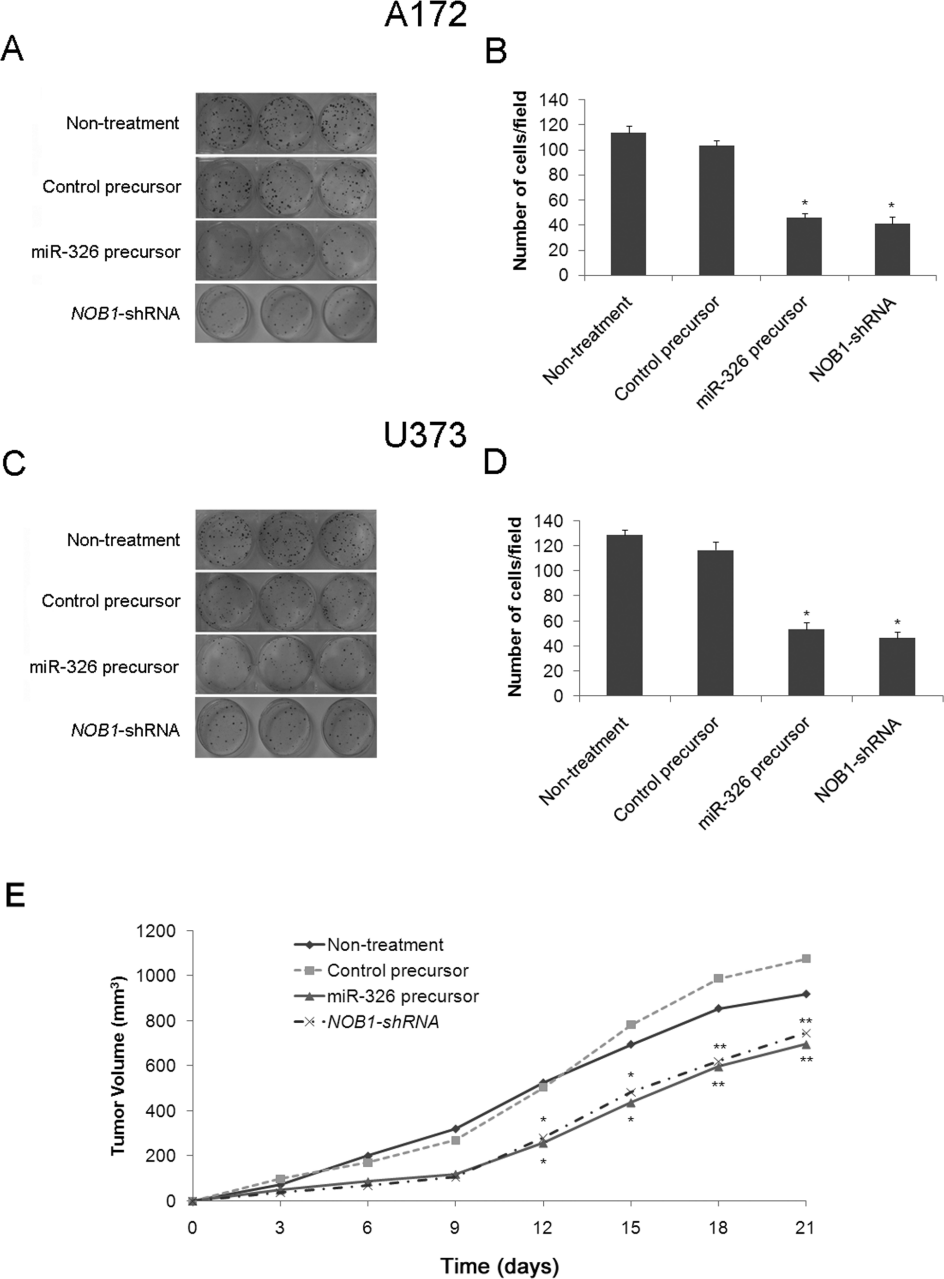
*In vitro* and *in vivo* tumorigenesis of A172 and U373 cells treated with miR-326 precursor. (A, B) Soft agar assays were performed to investigate the effects of miR-326 on tumorigenesis *in vitro*. A172 cells that had been infected with miR-326 precursor, control precursor, *NOB1* shRNA or nothing were plated in wells coated with agar. After 3 weeks at 37°C, the colonies were stained and counted. (C, D) The same assay was performed on U373 cells. Data represent mean ± SD of three independent experiments. Significant differences between transfected cells and mock-infected cells were determined using the two-tailed Student’s *t*-test (*P<0.05). miR-326 overexpression or NOB1 silencing decreased colony formation. (E) A mouse xenograft model was used to examine the effect of miR-326 on tumorigenesis *in vivo*. The tumors were measured in two dimensions using a caliper on different days. The volume (mm^3^) was calculated using the formula V = 0.5* larger diameter *(smaller diameter)^ 2^. miR-326 overexpression or *NOB1* shRNA significantly inhibited tumor formation. Data represent mean ± SD of three independent experiments. Significant differences between transfected cells and mock-infected cells were determined using one-way ANOVA (*P<0.05, **P<0.01).

To determine the role of miR-326 in the tumorigenesis of human glioma cells *in vitro*, colony formation in soft agar was assessed in A172 and U373 cells. Similar to *NOB1* silencing, overexpression of miR-326 caused a substantial reduction in colony formation in soft agar compared with the control cells (P<0.05; Figures 5A–D). To further confirm the effect of increased miR-326 levels on the tumorigenesis of glioma cells *in vivo*, U373 cells transfected with miR-326, miR-control or *NOB1* shRNA were inoculated into the flank of nude mice. The changes in tumor volume were monitored for 3 weeks. Mean tumor volume in the miR-326 group or the *NOB1*shRNA group was 697.02 mm^3^ or 745.91 mm^3^, whereas tumor volumes in mice treated with saline or negative control plasmid were 919.56 mm^3^ or 1077.27 mm^3^, respectively (P<0.01) after 21 d (Figure 5E). These results demonstrated that miR-326 overexpression significantly inhibited tumor formation in human glioma cells, and suggest that miR-326 may play a critical role in the tumorigenicity of human glioma cells *in vitro* and *in vivo*.

### miR-326 Regulates the Expression of Key Components of the MAPK Signaling Pathway

**Figure 6:**
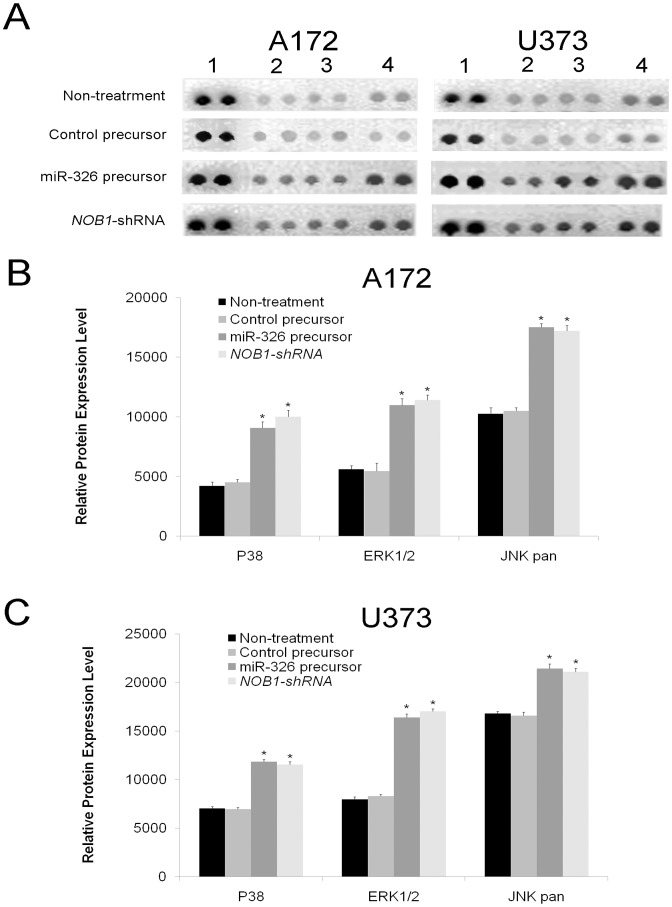
Overexpression of miR-326 alters the expression of key components of the MAPK pathway. (A) Whole-cell extracts of A172 and U373 glioma cells with different treatments were incubated on a Phospho-Kinase Array and phosphorylation status was determined by subsequent incubation with anti-phosphotyrosine horseradish peroxidase. Each protein was spotted in duplicate. (1. Positive control; 2. P38; 3. ERK 1/2; 4. JNK pan). In A172 (B) and U373 (C) cells, the phosphorylation of all three components of MAPK pathway were significantly increased after miR-326 overexpression or *NOB1*-shRNA compared to the controls. Data represent mean ± SD of three independent experiments. Significant differences among groups were determined using one-way ANOVA with LSD method (*P<0.05).

The phosphorylation of several proteins was analyzed using the Phospho-Kinase Array Kit. In U373 and A172 cells, the phosphorylation of three members of the MAPK pathway, p38 (T180/Y182), ERK1/2 (T202/Y204, T185/Y187), and JNK (T183/Y185, T221/Y223) increased significantly after NOB1 suppression compared to the negative control (P<0.05, Figure 6).

### Overexpression of *NOB1* in Glioma Tissue Samples and its Correlation with Poor Prognosis

**Figure 7:**
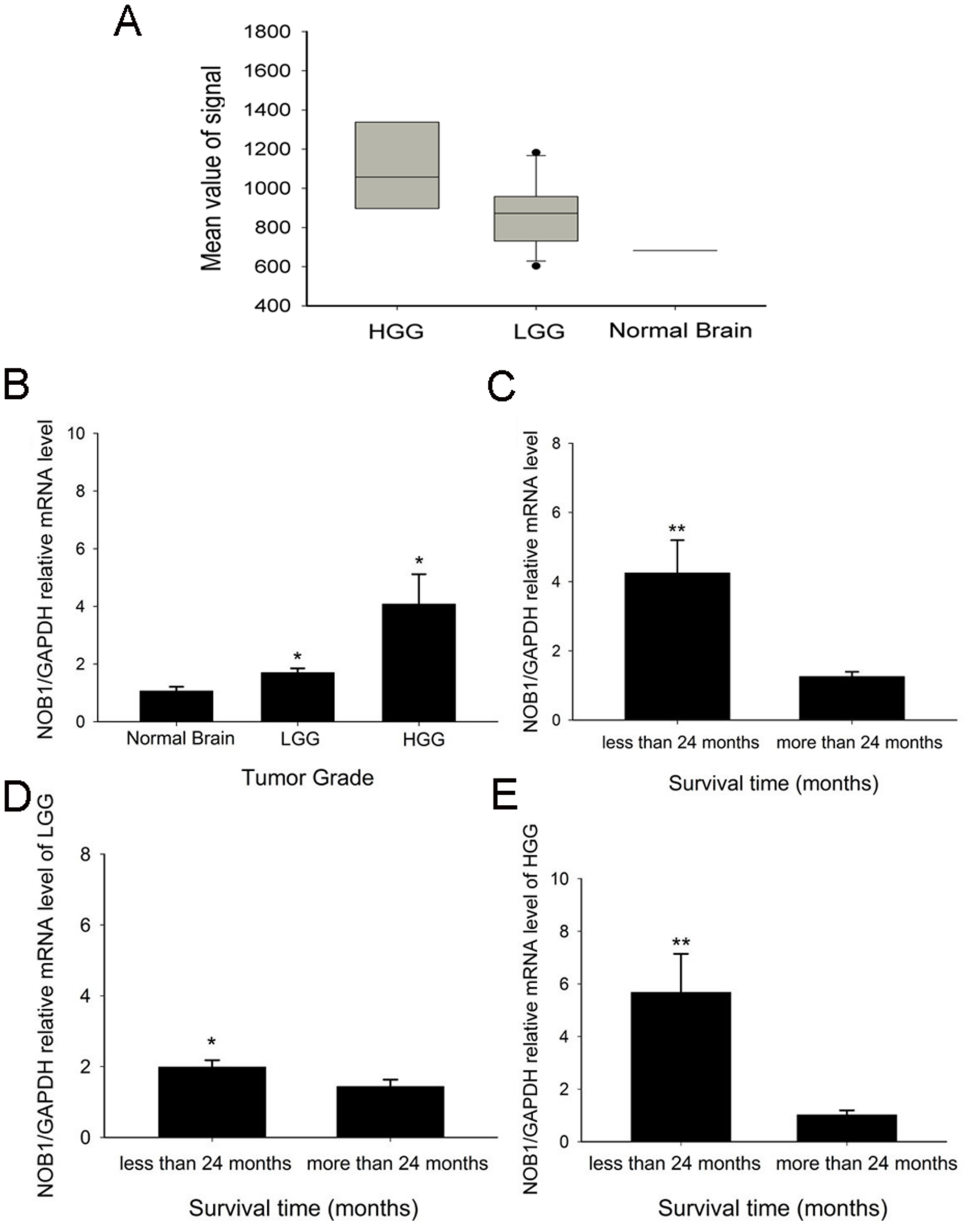
NOB1 expression examined by Affymetrix Genechip, and mRNA and protein levels in glioma samples. (A) The expression signal corresponding to *NOB1* was significantly higher in high-grade glioma samples compared with low-grade glioma (P = 0.01) and normal brain samples (P<0.001), although the difference between low-grade glioma and normal brain was not statistically significant (P = 0.100). Differences between groups were assessed by one-way ANOVA with the LSD method (*P<0.05). (B) Quantitative RT-PCR showed that *NOB1* mRNA was up-regulated in low grade glioma (LGG) and high grade glioma (HGG) tissue samples (P = 0.017 and P = 0.032, respectively) compared with normal brain tissue samples from 7 volunteers. (C) Glioma patients who lived more than 24 months (23 patients, 41.8%) showed decreased *NOB1* mRNA expression, whereas patients who lived less than 24 months (32 patients, 58.2%) showed higher *NOB1* mRNA expression (P<0.01) regardless of glioma grade. (D) In patients with LGG, those who lived more than 24 months (13 patients, 52%) showed lower *NOB1* mRNA expression, whereas those who lived less than 24 months (12 patients, 48%) showed higher *NOB1* mRNA expression (P = 0.028). (E) In patients with HGG, those who lived more than 24 months (10 patients, 33%) showed lower *NOB1* mRNA expression, whereas those who lived less than 24 months (20 patients, 67%) showed higher *NOB1* mRNA expression (P<0.01). The relative expression of *NOB1* mRNA in each group was expressed as mean ± SE, and the differences between groups were determined using the Mann-Whitney U test (*P<0.05. **P<0.01).

**Figure 8:**
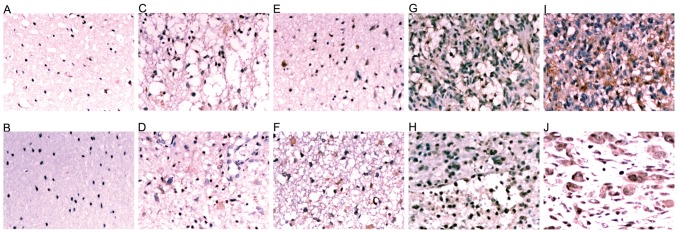
Expression of NOB1 protein in glioma and normal brain tissue samples. Immunohistochemical staining of normal brain tissue (A, B), grade I (C, D), grade II (E, F), grade III (G, H) and grade IV (I, J) glioma tissue specimens expressing NOB1. NOB1 staining was stronger in high-grade gliomas than that in low-grade gliomas. No significant staining was observed in normal brain tissues.

Analysis of *NOB1* expression using an Affymetrix GeneChip showed that the expression of *NOB1* in high-grade glioma was significantly higher than in low-grade glioma and the normal brain (P<0.001 and P = 0.01, respectively, Figure 7A). This result was confirmed by real-time polymerase chain reaction (RT-PCR) and immunohistochemistry. As shown in Figure 7B, the expression of *NOB1* mRNA in high-grade gliomas was approximately 4-fold (P = 0.017) higher, and that of low-grade gliomas was 1.5-fold (P = 0.032) higher than that of normal brain tissues. The difference of *NOB1* expression between high-grade gliomas and low-grade gliomas was also significant (P = 0.049). Moreover, immunohistochemical detection of NOB1 in glioma tissues (Figure 8) showed stronger staining for NOB1 in high-grade glioma than in low-grade glioma samples (P = 0.003), whereas normal brain tissues did not show significant NOB1 staining. The association between NOB1 expression and tumor grades in glioma strongly suggested that NOB1 might be involved in human brain tumorigenesis.

We then tested the correlation between NOB1 expression and the prognosis of glioma patients. Among the glioma patients analyzed, those who lived longer than 24 months (23 patients, including 13 LGG patients and 10 HGG patients) showed lower expression of NOB1, while those who lived less than 24 months (32 patients, including 12 LGG patients and 20 HGG patients) showed relatively higher expression of NOB1 (P<0.01, Figure 7C). Similar results were obtained after stratification of patients into low-grade glioma (P = 0.028; Figure 7D) and high-grade glioma (P<0.01; Figure 7E). These results indicated that higher levels of *NOB1* mRNA are associated with a relatively shorter survival.

## Discussion

Malignant glioma remains the most common and fatal brain tumor world-wide. In addition to conventional therapeutic strategies, targeted therapies are currently being developed to interfere with the transduction of key signaling pathways [18] or to inhibit the function of tumor specific molecules [19] in malignant glioma. It is widely accepted that the future treatment options for GBMs will greatly benefit from our improved understanding of the complex molecular mechanism in glioblastoma.

MicroRNAs are critical post-transcriptional regulators of several genes. Previous studies have suggested that the dysregulation of miRNAs may play an important role in cancer progression [7], [20]. Changes in miRNA profiling are associated with almost all aspects of cancer biology, including cell proliferation, migration and angiogenesis [21]. The development of targeted therapies using miRNAs as a novel and specific diagnostic and therapeutic tool has generated considerable interest. In the present study, we focused on miR-326, which has been shown to suppress tumor growth in medulloblastoma and malignant glioma. The down-regulation of miR-326 in gliomas was shown to be associated with a feedback loop involving Notch that impaired glioma cell tumorigenicity [11].

In this study, we demonstrated that miR-326 inhibits tumorigenesis both *in vitro* and *in vivo* by blocking a novel miR-326 target, NOB1, which interacts with the 19S regulatory particle and is required for the maturation of the 26S proteasome [22], [23]. Analysis of cell cycle distribution in human glioma cells overexpressing miR-326 showed a substantial decrease in S-phase and an increase in G1 phase populations, leading to a significant delay of proliferation in U373 and A172 glioma cells. This growth inhibitory effect was also observed by colony formation in soft agar and nude mouse xenograft assays, suggesting that miR-326 and NOB1 are critical for human glioma tumorigenesis *in vitro* and *in vivo*. Moreover, assessment of NOB1 levels in human glioma tissue samples showed the up-regulation of NOB1 expression. The results showing up-regulated expression of NOB1 in human brain samples together with the malignancy of glioma and associated short survival suggested that NOB1 may play a role in the development of glioma. Our results are supported by published datasets in Oncomine (www.oncomine.org). In the dataset of Sun Brain, NOB1 was over-expressed in diffuse astrocytoma, oligodendroglioma, anaplastic astrocytoma and glioblastoma compared to the normal brain. In the data set of French Brain, NOB1 was over-expressed in anaplastic oligodendroglioma and anaplastic oligoastrocytoma compared to the normal brain. These data support the involvement of NOB1 in the tumorigenesis of glioma.

The present results showed that NOB1 is highly expressed in glioma cell lines and tissues, whereas its expression is decreased in normal brain tissue. These findings suggest the therapeutic potential of NOB1 inhibition for glioma. Moreover, the expression of NOB1 might be associated with tumor grades as well as the prognosis of glioma patients.

The activation of the MAPK pathway has been associated with glioblastoma proliferation, the rapid growth of which is responsible for the lethal nature of this tumor [18]. We hypothesized that the induction of early apoptosis by NOB1 down-regulation in glioma cells might be related to the MAPK signaling pathway. MAPK signaling is mediated by ERK1/2, JNK and p38 MAPK, which are important in the control of cell proliferation, differentiation and apoptosis [24], [25], [26]. Our results showed that silencing of NOB1 expression increased the phosphorylation of these 3 proteins, suggesting that the anti-glioma effect of NOB1 might be mediated by MAPK activation.

**Figure 9:**
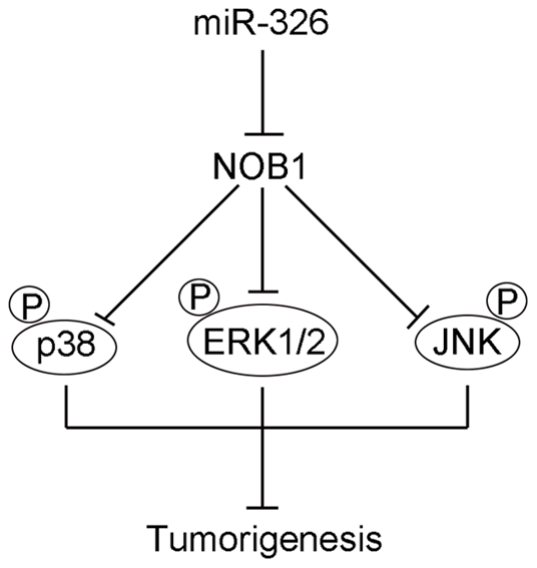
Schematic diagram illustrating the interplay among miR-326, NOB1 and the MAPK pathway in glioma. miR-326, as a tumor suppressor by targeting NOB1, decreased the tumorigenesis of glioma cells *in vivo* and *in vitro* through the modulation of the MAPK pathway. Overexpression of miR-326, which suppresses the expression of NOB1, activates the MAPK patheay by increasing the phosphorylation of ERK1/2, JNK and p38 MAPK, which inhibits the cell proliferation and induced apoptosis in human glioma.

In conclusion, NOB1 was identified as a novel target of miR-326. Overexpression of miR-326 decreased the tumorigenesis of glioma cells *in vivo* and *in vitro* through the modulation of the MAPK pathway. The interplay among miR-326, NOB1 and the MAPK pathway was shown in Fig. 9. Moreover, NOB1 expression might be associated with tumor grade as well as the prognosis of glioma patients. These findings indicate that exogenous overexpression of miR-326 may prove to be a promising strategy for targeted therapies in malignant glioma.
